# Craniofacial Pain as the Sole Sign of Prodromal Angina and Acute Coronary Syndrome: A Review and Report of a Rare Case

**DOI:** 10.7508/iej.2015.04.013

**Published:** 2015

**Authors:** Mahta Fazlyab, Ehsan Esnaashari, Mojgan Saleh, Farshad Shakerian, Davood Akhlagh Moayed, Saeed Asgary

**Affiliations:** a *Iranian Center for Endodontic Research, Research Institute of Dental Sciences, Dental School, Shahid Beheshti University of Medical Sciences, Tehran, Iran; *; b *Department of Endodontics, Dental Branch, Islamic Azad University of Medical Sciences, Tehran, Iran;*; c *Dentist,Tehran, Iran; *; d *Interventional Cardiologist,Shahid Rajaie Cardiovascular, Medical and Research Center, Tehran, Iran; *; e *Interventional Cardiologist,Head of Cardiac Catheterization Laboratory, Pars Hospital, Tehran, Iran*

**Keywords:** Craniofacial Pain, Myocardial Infarction, Orofacial Pain, Pre-Infarction Angina, Prodromal Angina, Referred Pain

## Abstract

Orofacial pain can arise from different regions and etiologies. Some of the most debilitating pain conditions arise from the structures innervated by the trigeminal system (head, face, masticatory musculature, temporomandibular joint and associated structures). The problem with referred pain is the misdiagnosis and unnecessary therapy directed to the pain location instead of its origin. When craniofacial pain is the sole sign of myocardial ischemia, failure to recognize its cardiac source can endanger the patient. In particular, apart from unnecessary dental treatments, patients with acute myocardial infarction who do not experience chest pain run a very high risk of misdiagnosis and death. As endodontists, each of us may face many patients complaining of pain sensation in the teeth with the main source being other craniofacial/visceral organs. This review plots a diagnostically challenging case paving the way for further literature presentation in this regard. The aim of this compendious review was to gain knowledge about the prevalence, clinical characteristics and possible mechanisms of craniofacial pain of cardiac origin, in order to improve the clinician’s ability to make a correct diagnosis.

## Introduction

Orofacial pain disorders are prevalent conditions involving the head, face and neck. The orofacial region is complex and therefore, pain can arise from many sources [1]. The sensation of pain in a location distant from its source, *aka* the referred pain or heterotopic pain, is a common challenge to clinicians [2]. In the craniofacial area, this phenomenon can have several well-documented sources such as odontogenic, myofacial and temporomandibular joint [3]. As its name suggests, non-odontogenic toothache, is a pain that occurs in the absence of clinical evidence in the teeth or their surrounding structures [4]. It is estimated that among patients who appear in dental office due to toothache, 3% have non-odontogenic pain source and 9% have a mixed condition with both odontogenic and non-odontogenic causes [5]. While the diagnosis/treatment of primary toothache is not difficult, studies on non-odontogenic toothache is not routine. In fact the common approach to this phenomenon is conducting unnecessary, ineffective and sometimes irreversible dental treatments (pulpectomy or extraction) [5, 6]; in other words patients may suffer from long-lasting pain with unknown origin even after dental treatment [4]. 

As outlined by Okeson [7], the orofacial pain is classified into physical (Axis 1) and psychological (Axis 2) conditions. Physical conditions include disorders of the musculoskeletal structures for *e.g*. temporomandibular joint (TMJ), masticatory muscles and cervical spine; neuropathic pains which include episodic (*e.g.* trigeminal neuralgia) and continuous (*e.g*. peripheral/centralized mediated) pains and neurovascular disorders (*e.g.* migraine). Psychological conditions include mood and anxiety disorders.

According to a systematic review, apart from psychogenic toothache, primary mechanisms that lead to sensation of non-odontogenic craniofacial pain (CFP) fall into two main categories: *i)* projected nerve pain including neuropathic toothache and neurovascular pain and *ii)* referred pain as a result of convergence and central sensitization such as myofacial pain, idiopathic toothache, sinus pain and cardiac pain referred to the craniofacial area [4]. Dentists must to be aware of the possibility of ischemic heart disease in patients who visit the clinic with complaints of toothache only; almost 40% of ischemic heart disease patients experience facial pain during heart attacks with a significantly higher tendency in women while 15% experienced facial pain only [4]. 

A cross-sectional study on patients with acute coronary syndrome (ACS) was conducted 2014 [8]. Volunteers that were diagnosed with ACS during the past week by an interventional cardiologist, were questioned about the primary signs/symptoms. Patients with extensive decays were excluded due to the possibility of true concomitant presence of CFP with dental origin. For all volunteers the vitality of all teeth were tested with electric pulp tester (Analytic Technology, Redmond, WA, USA) and cold test with Green Endo Ice (Colten, Whaledent, NJ, USA) for 15 sec. The temporomandibular joint (TMJ) was carefully assessed to rule out the CFP secondary to TMJ disorders in each patient. The patients with definitely diagnosed ACS, were questioned about the location, quality, intensity, frequency and duration of pain as well as its elevating and relieving factors. All answers were recorded meticulously. The relation between the presence of MI on occurrence of CFP were evaluated using the multiple binary logistic regression model. Among 150 volunteers (54.7% male and 45.3% female), in 23 patients (15.3%) ACS represented with CFP which was bilateral in 18 (78.3%). Among them, 22 patients had experienced pain in the chest and left arm and shoulder while 15 reported neck pain along with CFP. Only one (4.3%) patient reported CFP as the only sign of ACS. There was a significant tendency for female patients to experience CFP; the similar tendency was observed for non-smoker and non-diabetic patients to report CFP as a sign of ACS.

ACS consists of variable clinical signs and symptoms related to acute myocardial ischemia (insufficient blood flow to the heart muscle as a consequence of narrowing or obstruction of a coronary artery that leads to imbalance between myocardial oxygen supply and demand) [9]. If myocardial ischemia is intense or prolonged, it can lead to cardiac cell death and release of biomarkers of myocardial infarction (MI) such as troponin *I* and troponin *T*, which can be detected by specific laboratory testing [10]. According to clinical manifestations, electrocardiography (ECG) and serum levels of biomarkers, ACS can be divided in two categories including unstable angina (new or changing symptoms in a crescendo pattern that can also occur at rest, contrary to stable angina) and MI [11]. A commonly forgotten manifestation of ACS is *prodromal (pre-infarction) angina* that will be addressed later.

Understanding the clinical characteristics of MI is a key factor in prompt and accurate diagnosis. Evaluating the pain location, quality, intensity, and duration, taking into account any aggravating, relieving and radiating factors, can be helpful in this regard [10]. Typically, general public recognize the cardiac pain as a localized pain in the sternal region and left side of the chest; however, it can also radiate to the neck, either arm, the shoulders, the stomach and the jaws [12]. The presence and intensity of pain is variable but is *not* associated with disease severity; both symptomatic and asymptomatic episodes can present with similar cardiac hemodynamic changes [13]. 

Patients who experience CFP as the sole symptom of myocardial ischemia are likely to seek dental treatment and thus the possibility of misdiagnosis is high. Until the date, the clinical link between CFP and myocardial ischemia was limited to case reports [14-18]. Toothache, mandibular pain, ear pain and headache were the most common reported pain locations. Misdiagnosis, mistreatment and delay in administration of appropriate therapy were common features in those reports [13, 16-19].

Although coronary disease is the leading cause of death in developed countries, its characteristics are not fully understood [19]. This fact increases the risk of clinicians and the general public being unaware of some atypical presentations; CFP is not usually mentioned either by physicians or by nurses as the only symptom that would suggest an ACS diagnosis [20]. As a result, significant number of patients with atypical symptoms die before receiving appropriate hospital care due to missed diagnosis and treatment delay [21].

There are several case reports of CFP as the one and only symptom of myocardial ischemia [14, 21, 22]. However, the frequency of myocardial ischemic pain being referred to craniofacial area has not been comprehensively studied in dental and cardiovascular research [17]. The presented case was one of many cases with referred pain sensation in the face stemming from myocardial ischemia that if was not diagnosed in time, would endanger patient’s life. In the present manuscript the prevalence, clinical characteristics and possible mechanisms of CFP of cardiac origin, are discussed in order to improve the clinician’s ability in making a correct diagnosis. 

The overall aim of this review is to provide clinical and diagnostic understanding of CFP of cardiac origin, with a special focus on CFP as the sole symptom of acute myocardial infarction (AMI) or a pre-infarction ischemic episode.

**Figure 1:**
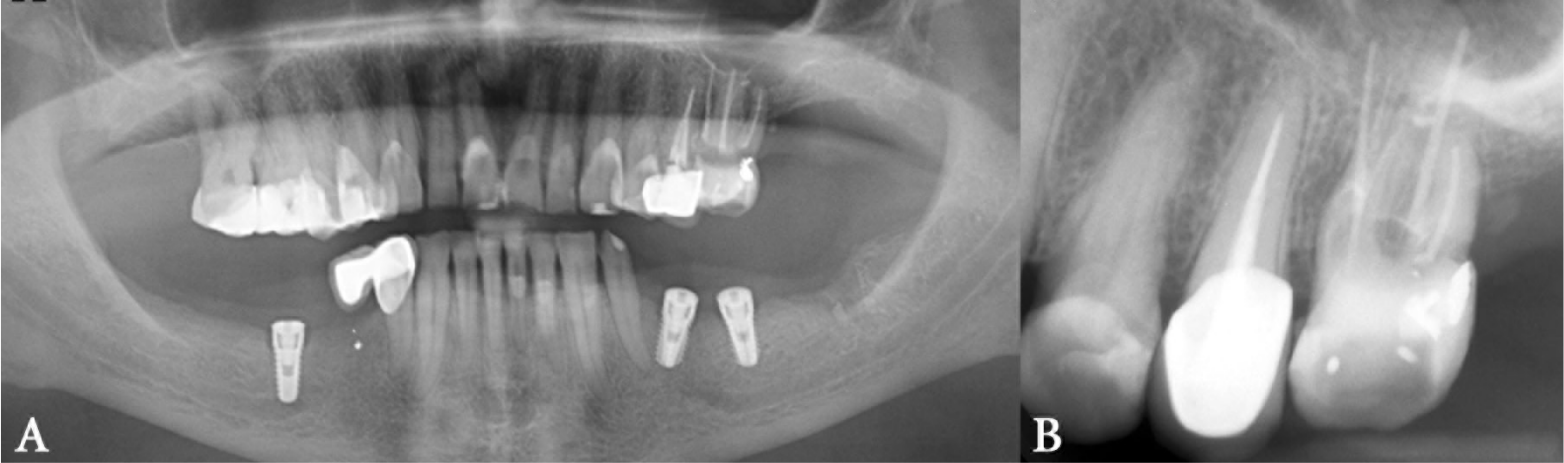
*A) *Panoramic image of the patient with the chief complain being dull pain in the left side of the face; tooth #26 was sensitive to percussion. *B) *Periapical view; endodontic treatment of tooth had been done five weeks earlier

## Case report

An elderly lady in her late fifties, was referred to the endodontic department of a private dental clinic with her chief complain being dull tight pain on the left side of her face. She stated that she had been experiencing this sensation of pain for the past two weeks. On clinical inspection no facial discrepancy and swelling was noticed. Oral examination revealed a maxillary first molar on the left side that had an extensive amalgam filling and was extremely sensitive to percussion and palpation of the periradicular area. She mentioned previous root canal therapy of this tooth five weeks earlier. Radiography was indicated and revealed a phenomenon that was supposed to be the cause of all these discomforts (Figures 1A and B). The mesiobuccal root was perforated in many zones and the perforation sites were over obturated with gutta-percha. Surprisingly she was told that her tooth had five canals and was extra-charged for these canals.

The situation was discussed with the patient and extraction/implant placement was indicated. Patient had too much anxiety and refused to accept the situation stating that she could not take that due to recent complications in her personal life. She insisted on an alternative approach. Thus, she was referred to another endodontist for evaluation of possibility of intentional replantation. It was later decided to approach through trial and error; if the replantation was not successful the tooth would be extracted. On the very same session the procedure was initiated. Unfortunately, the mesial root was separated during extraction and the clinician had to extract the tooth. 

One week later, she appeared with persisting pain on the left side of her face. This time she mentioned that this pain worsened when she became more stressful. She was not satisfied because she expected the pain would vanish after losing one tooth. As she was explaining her disapproval, it was observed that her face was blushing and she was sweating allover. The patient also mentioned pain worsening at the very same time. The clinician suggested an immediate medical visit because the manifestations seemed so irrelevant to odontogenic signs. One week later her daughter came to the dental office to inform that her mother was diagnosed with cardiac ischemia and myocardial infarction (MI) and expressed her gratitude for in time referral of her mother.

In summary, the primary cause of patient’s discomfort was CFP with cardiac origin (prodromal angina) not her tooth, whatever the condition of that tooth was. The in-time referral helped in saving patient’s life.

## Discussion

***Mechanisms of pain perception in the craniofacial region***

**Figure 2:**
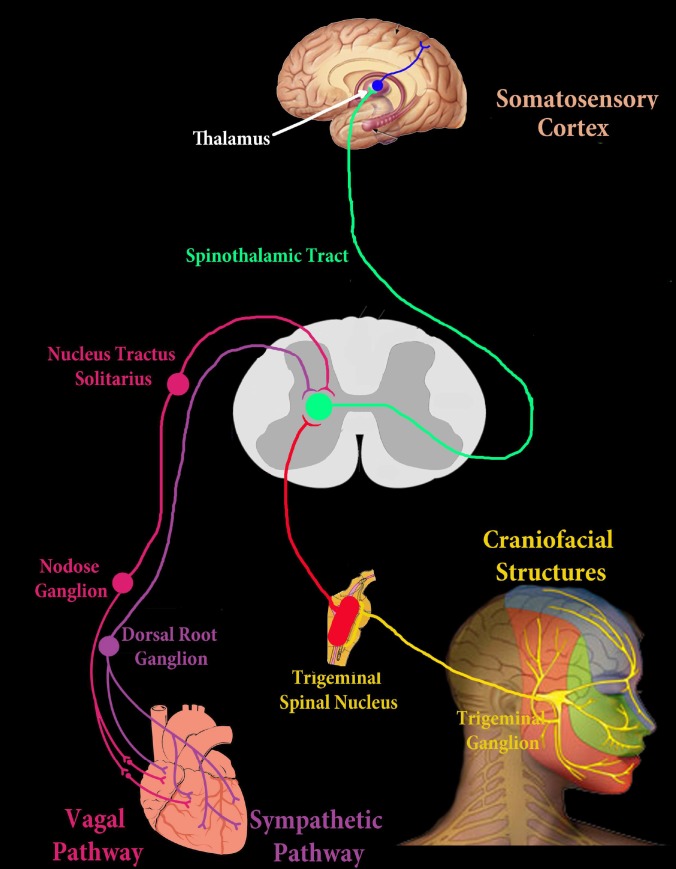
Schematic mechanism explaining the referral of cardiac pain to craniofacial structures (GG: Gasserian ganglion, NG: Nodose ganglion, DRG: Dorsal root ganglion, TSN: Trigeminal spinal nucleus, NTS: Nucleus tractus solitaries

The 5^th^ cranial nerve (trigeminal nerve-TG) is mainly responsible for the majority of pain sensations in the facial area through a very complex neurophysiology and peripheral/ central mechanisms [1, 18]. Each of the three branches of TG [including V1 (ophthalmic), the V2 (maxillary) and the V3 (mandibular)] nerves comprise of myelinated (*Aβ* and *Aδ*) and non-myelinated (*C*) fibers [21]. Nociceptive input is transmitted *via* *Aδ* and *C* fibers that are responsive to neural mediators [Substance P and calcitonin-gene related peptide (CGRP), neuropeptide Y, *etc.*] and are also activated by inflammatory mediators such as bradykinin and prostaglandins [23, 24]. Activation of the free endings of nociceptors involve complex biochemical interactions through membrane receptors or channels including the G-protein-coupled receptor (GPCR), the sodium channels, the voltage-gated potassium channels and the calcium channels [23]. Once the nociceptors (*Aδ* and *C* fibers) are activated, the afferent fibers conduct the action potentials to their cell bodies located in the trigeminal (*aka*. the Gasserian) ganglion. Then their axons conduct the nociceptive input to the starting point of central nervous system (CNS) (brainstem) and the trigeminal spinal nucleus (TSN) [21]. The most caudal zone of TSN is called sub-nucleus caudalis (located in the medullar dorsal horn) where the primary nociceptive afferent neurons of TG synapse with the second order neurons (Figure 2). 

The involved neurotransmitters in this synapse are glutamate, substance P and CGRP along with several modulatory circuits [25]. From the sub-nucleus caudalis, the second order (projection) neurons project the input to several sites within the central nervous system, including the thalamus that act as a relay for ascending nociceptive inputs to the sensory cortex [26].

***Mechanisms of cardiac pain***

Myocardial ischemia can occur as a result of increased myocardial metabolic demand (including extremes of physical exertion and severe hypertension), decreased delivery of oxygen/nutrients (when a thrombus is superimposed on an ulcerated or unstable atherosclerotic plaque) or both [27].

Occlusion of the coronary artery can activate the cardiac nociceptive afferent fibers [17]. Not a specific mediator is known responsible for this pain transmission and it is likely that multiple mediators are released at the same time that may interact with one another [21]. Concentration of bradykinin, thromboxane, adenosine, potassium, histamine and prostaglandins change during MI [11, 28] with bradykinin and adenosine being the most important mediators of cardiac pain and angina pectoris [18]. Angina-like chest pain stimulated by intravenous injection of adenosine has been reported [21]. Histamine can contribute perception of cardiac pain during MI, through the H1 receptors [29]. Furthermore, bradykinin and thromboxane A2 interact to stimulate cardiac afferent endings that are sensitive to ischemia and as a result lead to synergistic afferent responses [11, 28].

There is a significant anatomic overlapping of sympathetic and vagal fibers which are more concentrated in the anterior and posterior walls of the heart, respectively [30]. Sympathetic afferent fibers contribute to most of the pain perception during MI. However, severing the sympathetic fibers, did not eliminate feeling the pain in the neck and jaws which means vagal fibers are also involved in perception of cardiac pain specially originated from posterior wall of heart [21]. The cell bodies of the sympathetic and cardiac vagal afferent fibers from the heart are located in the dorsal root ganglia and the ganglion nodosum (nodose ganglion), respectively (Figure 2). From here the main ascending systems which conduct afferent information to the brain, are the spinothalamic and the spinoreticular tracts [31]. 

***Cardiac pain referred to the craniofacial region***

Convergence mechanisms are involved in craniofacial pain, which originates in the heart. The upper cervical spine segments are convergence areas for trigeminal, visceral and phrenic inputs [31]. The trigeminal sub-nucleus caudalis, receives extensive convergence inputs from cutaneous, muscular and visceral afferents [32].

There is a connection between cardiac vagal afferents and trigeminal and trigemino-thalamic neurons; moreover, cardiac inputs have a modulatory action on the trigeminal system [21]. According to Qin *et al.* [33], 89% of the C1-C3 neurons that receive somatic inputs from the craniofacial structures, are also excited by cardiac nociceptive afferent fibers which can justify the referred pain to the face during MI. In other words, C1-C2 spinal neurons may act as an integrating center for cardiac nociceptive from both vagal and sympathetic afferent fibers [33]. However, vagal afferents from the heart contribute to craniofacial pain more commonly because somatic fields in the jaws and the neck were are shown to be more reactive to vagal than to sympathetic experimental electrical stimulation [31] (Figure 2). 

The quality of craniofacial pain from cardiac or dental origin is different, implying a high diagnostic validity. Patients describe referred cardiac pain as a *tight* and *burning* sensation while odontogenic toothache is *throbbing* and *tingling*. Considering the common characteristics of ACS (aggravation induced by exercise, improvement at rest, bilateral symptoms, *etc.*), careful elicitation of pain history and meticulous patient assessment can be helpful in diagnostic decision making [4]. In the aforementioned patient, aggravation of facial pain after getting more nervous during explaining her dissatisfaction for persistent pain, correctly pointed to the possibility of cardiac origin. An echocardiogram can be used to diagnose aortic dissection in such situations. 

***Prodromal angina***

Prodromal angina (also called pre-infarction angina) is one or several acute episodes of myocardial ischemia prior to an AMI, occurring several hours, weeks or months earlier [34, 35]. Early prodromal warnings of an AMI are not well known either by the public or by clinicians. Patients who suffered from prodromal angina before being hospitalized for AMI, reported that they had ignored these prodromal symptoms, whereas others sought medical/dental assistance but the condition was minimized, misdiagnosed or ignored by clinicians [21, 34]. Prodromal angina is a strong predictor of better survival and smaller infarct zone in the myocardium [13]. *Ischemic preconditioning* plays role in this phenomenon the mechanisms of which are complex and still not well understood. Involvement of several mediators including bradykinin, adenosine and opioids as well as signaling kinases and mitochondrial molecules of the myocardium, is suggested [17, 21, 34]. Until now, the inclusion criteria for clinical research on prodromal angina have included chest pain/discomfort with or without other symptoms [3, 35]. However, prodromal angina seems to be the reason for CFP in the presented case earlier. In fact what our patient had been experiencing during the past weeks, was prodromal angina not a common toothache, regardless of the dramatic situations of the maxillary molar. Thus, the possibility of prodromal angina presenting as CFP alone should not be overlooked.

Patients with angina pain referred to craniofacial area must be referred to the cardiologist or other internal medicine specialists for treatment of the primary disease. Treatment includes anti-angina drugs (*β*-adrenoreceptor blockers, nitric acid, *etc.*) and antithrombotic drugs (antiplatelet drugs, anticoagulants, *etc.*).

***Risk factors and diagnosis***

MI is the leading cause of death in the United States and in most industrialized nations throughout the world [36]. The incidence of MI increases with age and it is dependent on predisposing risk factors for atherosclerosis. Six primary risk factors have been identified with the development of atherosclerotic coronary artery disease and MI including hyperlipidemia (elevated levels of total cholesterol, LDL, or triglycerides), diabetes mellitus (through increasing the rate of atherosclerotic progression and adversely affecting the lipid profile) [37, 38], hypertension [39], tobacco use (increasing platelet thrombus formation in areas with high shear forces, such as stenotic vessels) [40], male gender, and family history of atherosclerotic arterial disease [41]. 

Identifying a patient who is currently experiencing an MI can be straightforward, difficult, or somewhere in between [42]. Acute MI can represent with unique manifestations in individuals. The symptoms ranges from none to sudden cardiac death [38]. Although an asymptomatic MI is not necessarily less severe than a symptomatic event, patients who experience asymptomatic MIs are more likely to be diabetic [27]. However, there are some characteristic symptoms that can be helpful: chest pain described as a pressure sensation, fullness, or squeezing in the midportion of the thorax, radiation of chest pain into the jaw or teeth, shoulder, arm, and/or back, associated dyspnea or shortness of breath, associated epigastric discomfort with or without nausea and vomiting (probably through vagal system), associated diaphoresis or sweating and impairment of cognitive function without other causes [27]. An MI can occur at any time of the day (mostly around the early hours of the morning or are associated with demanding physical activity, or both) [10, 27]. Approximately 50% of patients have some warning symptoms (angina pectoris or an anginal equivalent such as prodromal angina) before the infarct [27]. 

The goals of therapy in acute MI are the restoration of normal coronary blood flow and the maximum salvage of functional myocardium. The strategy includes prescription of antiplatelet agents [36], supplemental oxygen [41], intravenous nitrates [36], prompt and adequate pain control [10], *β*-blocker therapy and use of a fibrinolytic agent , *etc*. The primary obstacles to achieving these goals are the patient's failure to recognize MI symptoms quickly and the delay in seeking medical attention. 

## Conclusion

This review focused on the mechanisms of referral craniofacial pain with cardiac origin outlined the guidelines for in time diagnosis of acute coronary syndrome (unstable angina and acute myocardial infarction) with non-odontogenic craniofacial pain as one of the signs or the only announcement. Scientific knowledge should be addressed to all medical personnel involved in dental treatment. 
